# Facial Cosmetics Exert a Greater Influence on Processing of the Mouth Relative to the Eyes: Evidence from the N170 Event-Related Potential Component

**DOI:** 10.3389/fpsyg.2016.01359

**Published:** 2016-09-05

**Authors:** Hideaki Tanaka

**Affiliations:** Department of Psychology, Faculty of Psychology, Otemon Gakuin UniversityIbaraki, Japan

**Keywords:** N170, event-related potential, cosmetic makeup, eyes, mouth, face perception

## Abstract

Cosmetic makeup significantly influences facial perception. Because faces consist of similar physical structures, cosmetic makeup is typically used to highlight individual features, particularly those of the eyes (i.e., eye shadow) and mouth (i.e., lipstick). Though event-related potentials have been utilized to study various aspects of facial processing, the influence of cosmetics on specific ERP components remains unclear. The present study aimed to investigate the relationship between the application of cosmetic makeup and the amplitudes of the P1 and N170 event-related potential components during facial perception tasks. Moreover, the influence of visual perception on N170 amplitude, was evaluated under three makeup conditions: Eye Shadow, Lipstick, and No Makeup. Electroencephalography was used to monitor 17 participants who were exposed to visual stimuli under each these three makeup conditions. The results of the present study subsequently demonstrated that the Lipstick condition elicited a significantly greater N170 amplitude than the No Makeup condition, while P1 amplitude was unaffected by any of the conditions. Such findings indicate that the application of cosmetic makeup alters general facial perception but exerts no influence on the perception of low-level visual features. Collectively, these results support the notion that the application of makeup induces subtle alterations in the processing of facial stimuli, with a particular effect on the processing of specific facial components (i.e., the mouth), as reflected by changes in N170 amplitude.

## Introduction

Women in a number of societies throughout the world have traditionally used cosmetic makeup to modify the visual perception of their facial beauty (Jones and Kramer, 2015), a process that elicits enhanced ratings of physical attractiveness from both men and women (Graham and Jouhar, 1981; Ueda and Koyama, 2011). Such results indicate that cosmetic makeup significantly influences the visual perception of facial stimuli.

While faces consist of similar physical structures, cosmetic makeup is typically used to highlight or emphasize individual features (i.e., eyes, nose, and mouth). Indeed, research has indicated that such enhancements are particularly relevant for the eyes (i.e., eye shadow) and the mouth (i.e., lipstick) (Graham and Jouhar, 1981; Ueda and Koyama, 2011; Jones and Kramer, 2015). Application of cosmetics to female faces was found to increase facial contrast (Russell, 2009). According to Russell (2003), the “consistent luminance difference between the darker regions of the eyes and mouth and the lighter regions of the skin that surround them forms a pattern unique to faces.” Russell et al. (2016) also reported that female faces with higher facial contrast were rated as healthier and more attractive when this difference in luminance was increased than when it was decreased, though the opposite was observed for male faces (Russell, 2003). In addition, previous studies have indicated that increasing facial contrast via the use of cosmetics plays a role in age perception, and that female faces with greater facial contrast appear younger (Porcheron et al., 2013; Jones et al., 2015). Furthermore, female faces to which cosmetics have been applied are considered more feminine and attractive than the same faces without cosmetics (Russell, 2009; Stephen and McKeegan, 2010; Jones et al., 2015). Because increased luminance contrast enhances femininity and attractiveness in female faces, but reduces masculinity and attractiveness in male faces (Russell, 2003, 2009; Stephen and McKeegan, 2010), only female faces were utilized in the present study.

Changes in facial perception can be detected via the recording of event-related brain potentials (ERPs), from which studies have identified face-sensitive P1 and N170 components. As the P1 and N170 ERP components are typically regarded as markers for processing the perceived faces, these components are useful in examining the effect of cosmetics on face perception.

P1, an early positive component of the ERP, typically peaks approximately 100 ms after the presentation of facial stimuli. Reported to reflect the processing of low-level visual features such as contrast (Tarkiainen et al., 2002; Rossion and Caharel, 2011), P1 amplitude has also been linked to face-specific visual processing (Thierry et al., 2007; Susac et al., 2009; Dering et al., 2011; Luo et al., 2013). Previous studies indicate that P1 features a medial (O1, O2) or a lateral-occipital scalp distribution, or both (Eimer, 1998, 2000a; Liu et al., 2002; Goffaux et al., 2003; Itier and Taylor, 2004a,b; Herrmann et al., 2005; Okazaki et al., 2008; Sadeh et al., 2010; Luo et al., 2013).

Comparatively, N170 is a negative component evoked at the onset of facial perception that is characterized by a posterior-temporal scalp distribution (P7, PO7, PO8, P8) (Bentin et al., 1996; Rossion and Jacques, 2008; Chen et al., 2009; Peng et al., 2012; Ran et al., 2014) and peaks approximately 170 ms after the presentation of facial stimuli. N170 amplitude is significantly greater in response to human faces than other visual images, including cars, hands, houses, furniture, and scrambled faces (Bentin et al., 1996; George et al., 1996; Eimer, 1998, 2000b; Eimer and McCarthy, 1999; Jemel et al., 1999; Bentin and Deouell, 2000). The neural generators of N170 are reported to lie adjacent to the fusiform area, a region previously implicated in facial processing (Bentin et al., 1996). This is consistent with previous functional magnetic resonance imaging (fMRI), magnetoencephalography (MEG), and Brain Electrical Source Analysis (BESA) studies (Puce et al., 1995; Watanabe et al., 1999a,b; Sadeh et al., 2010; Luo et al., 2013).

While the P1 component of the ERP appears to reflect a response to lower-level visual features, the N170 component appears to be driven by wide-scale facial perception (Rossion and Caharel, 2011). In addition, Schweinberger (2011) suggested that the P1 component reflects the pictorial encoding of faces, while the N170 component reflects the structural encoding of faces. Pictorial encoding is defined as the early top–down attentional processing of faces, while structural encoding precedes the processes involved in the identification of faces. N170 is therefore considered to be related to both domain-specific and domain-general processing of facial information (Eimer, 2011). However, the ability of P1 or N170 amplitudes to detect alterations in facial perception induced by the application of cosmetics remains unclear. In order to clarify the influence of cosmetic makeup on the neural representation of facial stimuli, the present study used an ERP adaptation paradigm, in which the stimulus presented was preceded by a stimulus of another category (e.g., a face presented within a different format) (Kovács et al., 2006; Eimer et al., 2010; Zimmer and Kovács, 2011; Caharel et al., 2015). Since previous studies of cosmetic makeup have utilized comparisons between faces with and without makeup, (Graham and Jouhar, 1981; Jones and Kramer, 2015), the present study adopted a similar paradigm.

The current literature indicates that the eyes play a central role in facial perception and representation. In particular, a recent MEG study demonstrated that participants require significantly longer to perceive eyes presented in isolation than when presented as a facial component (Watanabe et al., 1999b). Accordingly, eye-tracking studies demonstrate that participants tend to fixate close to or directly on the eyes during facial perception (Janik et al., 1978; Barton et al., 2006; Arizpe et al., 2012), and that N170 amplitude is significantly greater in response to eyes presented in isolation than to the whole face, nose, or mouth (Bentin et al., 1996; Bentin and Deouell, 2000; Itier et al., 2006; Itier et al., 2007; Nemrodov and Itier, 2011). However, conflicting reports exist with regard to this matter. While several studies have indicated that eyeless faces elicit N170 amplitudes similar to that of normal or inverted faces (Eimer, 1998; Magnuski and Gola, 2013), a similar experiment demonstrated greater N170 amplitudes for normal faces relative to eyeless faces (Nemrodov et al., 2014). Therefore, the relationship between N170 amplitude and the role of eyes in facial perception remains uncertain.

In addition, daSilva et al. (2016) recently demonstrated the significance of the mouth in facial processing by presenting mouth images depicting grimaces, smiles, and open mouth expressions to participants. Expressions featuring teeth elicited significantly larger N170 amplitudes compared to expressions without teeth (daSilva et al., 2016). However, daSilva et al. (2016) did not examine N170 amplitude in relation to processing of both the eyes and mouth. Accordingly, few studies have evaluated N170 amplitude responsivity to the eyes and mouth in the context of facial processing. Pesciarelli et al. (2016) reported that processing of the eyes in inverted faces elicited significantly larger N170 amplitudes than in upright faces; however, this was not true for the mouth. In addition, Pesciarelli et al. (2016) identified significantly larger N170 amplitudes for the mouth relative to the eyes in upright faces.

Therefore, it remains unclear whether the eyes or mouth exert a greater influence on N170 amplitude. As previously mentioned, cosmetic makeup is most frequently applied to the eyes (i.e., eye shadow) and the mouth (i.e., lipstick) (Graham and Jouhar, 1981; Ueda and Koyama, 2011; Jones and Kramer, 2015). Mulhern et al. (2003) examined the relative contribution of cosmetic application under five cosmetic conditions: no make-up, foundation only, eye make-up only, lip make-up only, and full-facial make-up. Women judged eye make-up as contributing most to attractiveness, while men rated both eye make-up and foundation as having a significant impact on attractiveness in the context of a full-facial makeover. However, lipstick did not appear to contribute to attractiveness independently (Mulhern et al., 2003). On the other hand, Stephen and McKeegan (2010) allowed participants to manipulate the color of the lips in color-calibrated face photographs along the red–green and blue–yellow axes. Participants increased redness contrast to enhance femininity and attractiveness in female faces, but reduced redness contrast to enhance masculinity in male faces (Stephen and McKeegan, 2010). In order to clarify whether the application of cosmetics to the eyes or mouth elicits a greater effect on N170 amplitude, the present study compared P1 and N170 amplitudes during facial perception under three makeup conditions: *Eye shadow*, *Lipstick*, and *No Makeup*.

The present study aimed to investigate the influence of cosmetic makeup on the perception of facial stimuli via the evaluation of N170 and P1 amplitudes during the recording of ERP. If N170 amplitude is more reflective of the processing of the eyes than the mouth following the application of cosmetics, a significant difference in N170 amplitude would be expected between the *Eye shadow* and *Lipstick/No Makeup* conditions. Alternatively, if N170 amplitude is more reflective of the processing of the mouth than the eyes following the application of cosmetics, a significant difference would be expected between the *Lipstick* and *Eye shadow/No Makeup* conditions. Moreover, because the application of cosmetics to female faces has been observed to increase facial contrast (Russell, 2009), the present study also aimed to investigate whether application of cosmetics to the eyes or mouth exerts a greater influence on P1 amplitude, which reflects the processing of lower-level visual features (Rossion and Caharel, 2011).

## Materials and Methods

### Participants

Seventeen healthy, right-handed, Japanese participants (5 men; 12 women; aged 18–24 years; mean age: 21.3 years) were selected for the present study. All participants exhibited normal or corrected-to-normal vision and had no history of psychiatric or neurological disorders.

All participants provided written informed consent prior to participation in the study, in accordance with the Declaration of Helsinki. The ethics committee of Otemon Gakuin University formally approved this experiment and the recruitment of participants from the Otemon Gakuin University student population.

### Stimuli

**FIGURE 1:**
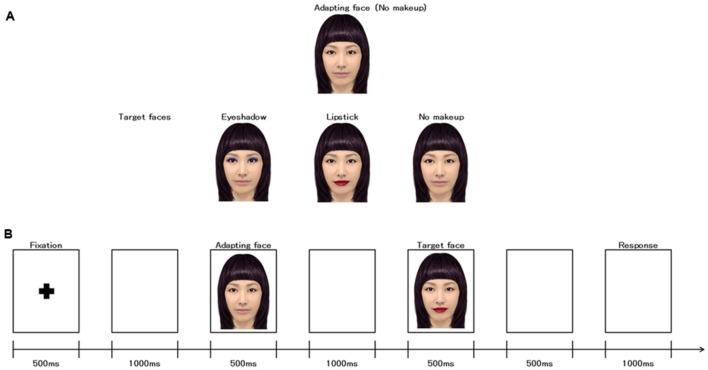
**(A)** Examples of adapting facial stimuli (*No Makeup*) and three target faces (*Eye shadow, Lipstick, No Makeup*). **(B)** Timeline of the single trial.

The images selected as visual stimuli included color pictures of the faces of 10 young, adult Japanese women. The stimulus faces were unfamiliar to all participants in the study. The pictures were obtained from various websites^1^^,^^2^ and included front-facing views of almost identical luminance. All images depicted neutral expressions. In total, 30 visual stimuli were used for the present study (each of the 10 model faces were provided in three conditions; *Lipstick* (wearing red lipstick only), *Eye Shadow* (wearing blue eye shadow only), and *No Makeup* (no makeup applied). Each image was digitally edited to include cosmetics of the same color (same red lipstick, same blue eye shadow) and reconstructed from the original using an application software (YouCan Makeup) of iPad^3^ (**Figure 1A**). The *No Makeup* condition was also used as the adapting image for the experiment and was presented prior to each stimulus for comparison. In addition, each image was edited to feature the same hairstyle and color (black), as reconstructed from the original using an iPad. All stimuli were airbrushed using Adobe Photoshop 12 to remove any outstanding features or blemishes and were subsequently processed using Photoshop software to ensure background consistency. All stimuli were presented in the same orientation on a white background. All faces were presented in a front-facing view and were equated for mean luminance (luminance values = 8.3 cd/m2) and size using Adobe Photoshop 12 software (**Figure 1A**). Faces occupied a visual angle of (horizontal × vertical) 3.4° × 4.0°. All faces were presented in the center of a 22-inch cathode ray tube monitor (Mitsubishi, Diamondtron M2, RDF223G, Chiyoda, Tokyo, Japan) that was placed 100 cm in front of the participants. The screen resolution was 1280 × 1024, with a refresh rate of 100 Hz.

A previous study (Golby et al., 2001) reported differential activity in the fusiform region in response to same-race and alternate-race faces. Comparatively, several reports indicate that N170 amplitude is unaffected by the effects of race (Vizioli et al., 2010a,b), while additional studies report greater N170 amplitudes in response to other-race facial stimuli relative to own-race stimuli (Stahl et al., 2010; Wiese et al., 2012). For this reason, the images used as visual stimuli in the present study were of Japanese women only, and all participants were Japanese. In addition, since N170 amplitude varies depending on the viewpoint of the face (Eimer, 2000b; Caharel et al., 2015), all faces were presented in a front-facing view. Furthermore, in several studies, N170 has been shown to be sensitive to emotional expression (Batty and Taylor, 2003; Eger et al., 2003; Caharel et al., 2005; Blau et al., 2007; Leppänen et al., 2007). Therefore, the faces used for visual stimuli in the present study featured neutral expressions.

### Procedure

Participants were seated comfortably 100 cm in front of a 22-inch cathode ray tube monitor on which stimuli were presented using a Multi Trigger System (Medical Try System, Kodaira, Tokyo, Japan). Each trial was completed as follows: (I) a fixation mark (+) was presented for 500 ms, followed by an inter-stimulus interval of 1000 ms; (II) an adapting facial stimulus (No Makeup) was presented for 500 ms, followed by an inter-stimulus interval of 1000 ms; (III) a target face stimulus (*Lipstick*, *Eye Shadow*, or *No Makeup*) was presented for 500 ms, followed by an inter-stimulus interval of 500 ms; and (IV) a judgment screen was presented for 1000 ms (**Figure 1B**). The inter-trial interval varied randomly between 500 and 1500 ms.

On the judgment screen, the three target faces were assigned a number: 1 = *Lipstick*; 2 = *Eye Shadow*; 3 = *No Makeup*. Each participant was instructed to compare the adapting face (No Makeup) with a target face and to identify the type of target face as quickly and accurately as possible. Participants were required to respond by pressing one of three buttons that corresponded to 1, 2, and 3 with their right index finger to indicate whether the facial stimulus belonged to the Lipstick, Eye Shadow, or No Makeup condition, respectively. Reaction time was measured using a digital timer accurate to 1 ms, beginning with the onset of stimulus presentation and finishing once participants had responded to the stimulus. Participants performed 10 practice trials, followed by three blocks of 80 trials (240 trials total). The order of conditions was randomized within each block. In total, 30 stimuli were presented in random order, with equal probability. In each trial, the adapting face and target face were obtained from the same person.

### Recording and Analysis

Electroencephalography (EEG) and Electrooculography (EOG) data were acquired using a 128-channel Sensor Net (Electrical Geodesic, Inc., Eugene, OR, USA) and recorded via the standard EGI Net Station 5.2.01 package. EEG and EOG results were recorded using Ag/AgCl electrodes from the 10-5 system (Oostenveld and Praamstra, 2001; Jurcak et al., 2007) and each electrode was referred to the vertex (Cz). Next, each electrode was oﬄine re-referenced to the common average. Vertical and horizontal eye movements were recorded using EOG electrodes placed above, below and at the outer canthi of both eyes to detect movement artifacts. EEG and EOG were sampled at 500 Hz and band-pass filtered at 0.01–30 Hz. Electrode impedance was maintained below 50 kΩ. For artifact rejection, all trials in which both the vertical and horizontal EOG voltages exceeded 140 mV during the recording epoch were excluded from further analysis.

Stimulus-locked ERPs were derived separately for each of the three target faces (*Eye shadow*, *Lipstick*, and *No Makeup*) from 200 ms before to 1000 ms after stimulus presentation, and were baseline corrected using the 200 ms pre-stimulus window. Based on previous studies (Luo et al., 2013; Ran et al., 2014), the P1 component was analyzed via the following four electrode sites: O1/O2 and PO3/PO4. The amplitude of the positive peak of the EEG signal was quantified 50–110 ms after stimulus presentation. Similarly, according to previous studies (Ran et al., 2014; Caharel et al., 2015), the N170 component was analyzed via the following 12 electrode sites: P5/P6, P7/P8, PO7/PO8, PO9/PO10, POO9h /POO10h, and PPO9h/PPO10h (the 10-5 system) (Oostenveld and Praamstra, 2001; Jurcak et al., 2007). The amplitude of the negative peak of the EEG signal was quantified 120 to 180 ms after stimulus presentation. The mean reaction time and ERP amplitude were then calculated for each participant in response to the three target faces.

### Statistical Analysis

Reaction time was analyzed using a one-way repeated-measures analysis of variance (ANOVA) for each condition (*Eye shadow*, *Lipstick*, *No Makeup*). P1 amplitude was analyzed using a three-way (3 × 2 × 2) repeated-measures ANOVA with regard to condition (*Eye shadow*, *Lipstick*, *No Makeup*), hemisphere (left, right) and electrode placement (O1 vs. PO3, O2 vs. PO4), while *post hoc* comparisons were performed using the Bonferroni test. Similarly, N170 amplitude was analyzed using a three-way (3 × 2 × 6) repeated-measures ANOVA with regard to condition (*Eye shadow*, *Lipstick*, *No Makeup*), hemisphere (left, right) and electrode placement (P5 vs. P7 vs. PO7 vs. PO9 vs. POO9h vs. PPO9h, P6 vs. P8 vs. PO8 vs. PO10 vs. POO10h vs. PPO10h), while *post hoc* comparisons were performed using the Bonferroni test. ERP was analyzed using Greenhouse–Geisser corrections applied to p values associated with multiple degrees of freedom repeated-measures comparisons.

## Results

### Effect of Condition on Reaction Time

No main effect was detected for *condition* on reaction time: *Eye shadow*, 408 ± 81 ms (mean ± SD); *Lipstick*, 412 ± 98 ms; *No Makeup*, 391 ± 73 ms [*F*(2,32) = 1.70, *p* = 0.20].

### Effect of Condition on P1 Amplitude

**FIGURE 2:**
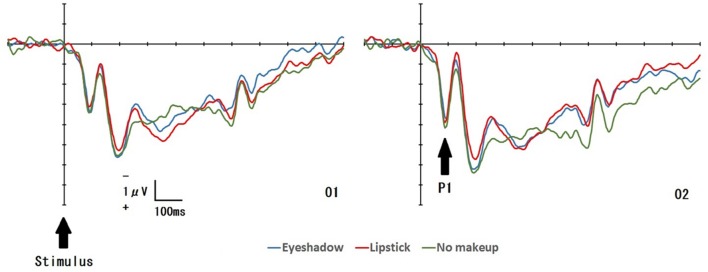
**Stimulus-locked average ERP waveforms at O1 and O2 for each target face: *Eye shadow*, *Lipstick*, and *No Makeup***.

**Figure 2** displays the grand-averaged EEG waveforms for all conditions (*Eye shadow*, *Lipstick*, *No Makeup*) at two electrode sites (O1/O2). For each condition, an enhanced positive ERP was identified 50–110 ms after exposure to the target face. This positive ERP was identified as P1. **Table 1** displays the mean P1 amplitude for all conditions at four electrode sites (O1/O2 and PO3/PO4). No significant main effect was detected with regard to *condition* [*F*(2,32) = 0.49, *p* = 0.77, $\eta_{p}^{2}$ = 0.02), or *hemisphere* on P1 amplitude [*F*(1,16) = 0.42, *p* = 0.53, $\eta_{p}^{2}$ = 0.03]. However, *electrode placement* produced a significant effect on P1 amplitude [*F*(1,16) = 11.95, *p* = 0.003, $\eta_{p}^{2}$ = 0.43], with a greater P1 amplitude at the O1 and O2 sites than the PO3 and PO4 sites (*p* < 0.05). No significant interactions were observed between the variables (all *p* > 0.05).

**Table 1:** Mean P1 amplitude for all conditions at four electrode sites.

Electrocode	Eyeshadow		Lipstick		No makeup	
	Mean	*SD*	Mean	*SD*	Mean	*SD*
**Left hemisphere**				
PO3	1.53	1.06	1.22	0.95	1.22	1.07
O1	2.17	1.56	2.15	1.88	2.21	1.98
**Right** **hemisphere**				
PO4	1.45	1.69	1.34	1.26	1.51	1.44
O2	2.40	1.33	2.43	1.26	2.69	1.67

### Effect of Condition on N170 Amplitude

**FIGURE 3:**
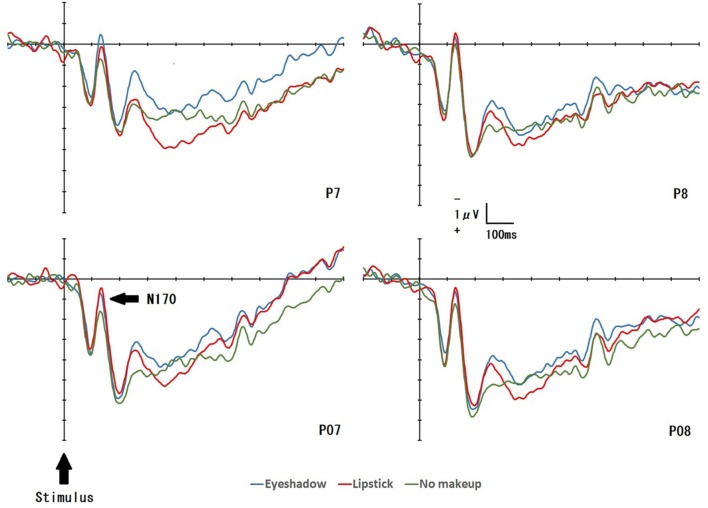
**Stimulus-locked average ERP waveforms at P7, P8, PO7, and PO8 for each target face: *Eye shadow*, *Lipstick*, and *No Makeup***.

**Figure 3** displays the grand-averaged EEG waveforms for all conditions (*Eye shadow*, *Lipstick*, *No Makeup*) at four electrode sites (P7/P8 and PO7/PO8). For each condition, an enhanced negative ERP was identified 120–180 ms after exposure to the target face. This negative ERP was identified as N170. **Table 2** displays the mean N170 amplitude for all conditions at 12 electrode sites (P5/P6, P7/P8, PO7/PO8, PO9/PO10, POO9h /POO10h, and PPO9h /PPO10h). A significant main effect was detected for *condition* [*F*(2,32) = 3.39, *p* = 0.05, $\eta_{p}^{2}$ = 0.18], and *electrode placement* on N170 amplitude [*F*(5,80) = 7.07, *p* = 0.002, $\eta_{p}^{2}$ = 0.31]. The N170 amplitude for the *Lipstick* condition was significantly greater than for the *No Makeup* condition (*p* < 0.05). No significant main effect was detected with regard to *hemisphere* on N170 amplitude [*F*(1,16) = 0.29, *p* = 0.60, $\eta_{p}^{2}$ = 0.02]. In addition, no significant interactions were detected between any two of the three variables (*condition* × *hemisphere*, *condition* × *electrode*, and *hemisphere* × *electrode*) (all *p* > 0.05), however, a significant interaction was detected for all three (*condition* × *hemisphere* × *electrode*) [*F*(10,160) = 2.88, *p* = 0.04, $\eta_{p}^{2}$ = 0.15]. Simple effect analyses indicated that the N170 amplitude for the *Lipstick* condition was significantly greater than for the *No Makeup* condition in the left hemisphere (PO7) and right hemisphere (PO10) (*p* < 0.05). Furthermore, simple effect analyses demonstrated that N170 amplitude was significantly greater at the P8 and PPO10h placement sites than the PO8 site in the right hemisphere for all conditions (*Eye shadow*, *Lipstick*, *No Makeup*) (*p* < 0.05). Moreover, in the left hemisphere during the *No Makeup* condition, N170 amplitude was significantly greater at P5 than P7 (*p* < 0.05).

**Table 2:** Mean N170 amplitude for all conditions at twelve electrode sites.

Electrocode	Eyeshadow		Lipstick		No makeup	
	Mean	*SD*	Mean	*SD*	Mean	*SD*
**Left hemisphere**				
P5	0.30	2.13	0.34	1.73	0.33	1.57
P7	1.04	3.56	1.48	2.60	1.87	2.53
PO7	2.52	4.32	2.17	3.54	3.14	4.14
PO9	0.73	2.68	0.66	2.33	1.11	2.24
POO9h	1.78	3.47	1.81	3.03	2.07	3.41
PPO9h	1.76	3.68	1.10	2.89	1.51	2.85
**Right hemisphere**				
P6	1.50	2.83	1.04	2.84	1.50	3.20
P8	1.24	4.23	1.85	3.95	2.32	4.31
PO8	3.06	4.30	2.74	3.71	3.52	4.79
PO10	1.04	3.13	0.77	2.61	1.66	3.35
POO10h	1.84	3.60	1.90	3.43	2.86	4.72
PPO10h	1.16	3.89	0.98	3.75	1.55	4.27

## Discussion

The present study aimed to investigate changes in P1 and N170 amplitude in response to the application of cosmetics during a facial perception task. This experiment adopted an ERP adaptation paradigm, in which participants were required to compare a model face with makeup (*Eye Shadow/Lipstick*) to a model face without makeup (*No Makeup*). Subsequently, P1 and N170 amplitudes were analyzed using EEG and EOG during a facial perception task with three target faces; (*Eye shadow*, *Lipstick*, and *No Makeup*), wherein the adapting face presented prior to the target face was the *No Makeup* condition. The results of the present study demonstrated that N170 amplitudes were significantly greater in response to the *Lipstick* condition than to the *No Makeup* condition, while no significant effect was detected for cosmetic makeup on P1 amplitude.

Previously, Rossion and Caharel (2011) reported that P1 and N170 amplitudes demonstrated functional dissociation with regard to facial sensitivity, wherein P1 was driven by low-level visual features, while N170 reflected facial perception. The present results support these findings, providing direct evidence that the application of facial cosmetics does not influence the perception of low-level visual features, but instead affects overall facial perception. Although the results of previous studies indicated that application of cosmetics to female faces increased facial contrast (Russell, 2009), no significant effect was detected for cosmetic makeup on P1 amplitude in the present study. As the present study utilized an ERP adaptation paradigm (Kovács et al., 2006; Eimer et al., 2010; Zimmer and Kovács, 2011; Caharel et al., 2015), attention was attracted to detecting changes between the faces with and without makeup. Therefore, because attention allocated to the low-level visual processing of faces was distracted, no differences in P1 were observed according to condition. On the other hand, because attention was allocated to the perception and structural encoding of the face, the N170 amplitude was significantly greater in the *Lipstick* than in the *No Makeup* condition.

Such findings provide support for the notion that the application of cosmetics significantly influences facial perception (Graham and Jouhar, 1981; Ueda and Koyama, 2011; Jones and Kramer, 2015). In the present study, N170 amplitudes were significantly greater in response to the *Lipstick* condition than to the *No Makeup* condition, though they did not significantly differ between the *No Makeup* and *Eye Shadow* conditions. Such findings support the hypothesis that N170 amplitudes reflect the processing of specific facial stimuli, wherein N170 better represents processing of the mouth than the eyes. Therefore, the results of the present study indicate that application of cosmetic makeup to any region of the face influences mouth-based processing, as reflected by changes in N170 amplitude. These findings are consistent with those of a previous study (Pesciarelli et al., 2016), wherein the application of cosmetic makeup (*Lipstick*) drew attention to the mouth during facial perception.

However, because different amounts of makeup affect attractiveness (Jones et al., 2014), it is possible that the red lipstick was more vivid than the blue eye shadow in the present study. In addition, because longer viewing durations affect judgments of faces with different amounts of makeup in varied ways (Etcoff et al., 2011), the red lipstick may have simply been more eye-catching under the relatively short duration utilized in the present study. To clarify these issues, future studies should manipulate the amount of makeup and duration of stimulus presentation.

In addition, Jones et al. (2015) revealed that a typical application of cosmetics increases the luminance contrast of the eyes to a much greater extent than the redness contrast of the mouth. The present results, however, contradicted these findings. As previously mentioned, because the present study did not involve manipulation of the amount of makeup or the duration of stimulus presentation, further research is required in order to examine the relative influence of various amounts of makeup on luminance contrast and ERP components. Furthermore, a previous eye-tracking study (Blais et al., 2008) revealed that Western Caucasian observers fixate more on the eye region, while East Asian observers fixate more on the central region of the face. It is possible that participants from a primarily Caucasian culture may not have exhibited the same pattern of results as the Japanese participants tested in the present study. Some previous studies indicate that N170 amplitude is unaffected by the effects of race (Vizioli et al., 2010a,b), while additional studies report greater N170 amplitudes in response to other-race facial stimuli relative to own-race stimuli (Stahl et al., 2010; Wiese et al., 2012). In order to clarify these issues, future studies should compare N170 amplitude for observers of several races when both own-race and other-race stimuli are presented.

According to Nemrodov et al. (2014), N170 amplitude was greater in response to inverted faces (faces presented upside down) than upright faces (Bentin et al., 1996; Rossion et al., 1999; Itier and Taylor, 2002; Pesciarelli et al., 2016). However, the N170 face inversion effect was strongly attenuated in eyeless faces when fixation was on the eyes, but was normal when fixation was on the mouth (Nemrodov et al., 2014). Moreover, Pesciarelli et al. (2016) reported that processing of the eyes in inverted faces elicited significantly larger N170 amplitudes compared to upright faces, though this effect was not observed for processing of the mouth. In addition, Pesciarelli et al. (2016) reported that processing of the mouth elicited significantly larger N170 amplitudes compared to processing of the eyes, but only in upright faces. Further research is needed to examine the relationship between the N170 face inversion effect and the application of cosmetic makeup, with particular focus on the eye and mouth regions.

While N170 amplitudes did not significantly differ between the *No Makeup* and *Eye Shadow* conditions in the present study, the eyes nonetheless play a significant role in facial perception (Janik et al., 1978; Bentin et al., 1996; Bentin and Deouell, 2000; Barton et al., 2006; Itier et al., 2006, 2007; Nemrodov and Itier, 2011; Arizpe et al., 2012; Nemrodov et al., 2014). Accordingly, Haxby et al. (2000) reported that the superior temporal sulcus processes individual facial features, including the changeable aspects of faces and the perception of eye gaze. Moreover, Cecchini et al. (2013) demonstrated that the left middle temporal gyrus (BA21) exhibits enhanced activation in response to eyes in an intact face condition than to eyes in a scrambled face condition. The results of the present study, in conjunction with the aforementioned findings, indicate that the role of the eyes in facial perception should be investigated with regard to other ERP components or using additional neuroimaging techniques.

## Conclusion

The present study found that N170 amplitude was significantly increased in response to the application of cosmetic makeup (*Lipstick*), but was unaffected by the *No Makeup* condition. In addition, no significant main effect was identified with regard to condition for P1 amplitude. Therefore, the present results support the notion that the application of cosmetic makeup alters facial perception. These findings subsequently indicate that cosmetic makeup produces a significant effect on facial perception, influencing the processing of specific facial features, with a particular focus on the mouth, as reflected by changes in N170 amplitude.

## Author Contributions

HT designed the experiments, performed the experiments and EEG data recording. HT performed the EEG data analysis and statistical analyses and wrote the manuscript.

## Conflict of Interest Statement

The author declares that the research was conducted in the absence of any commercial or financial relationships that could be construed as a potential conflict of interest.

## Footnotes

1 http://www.air-lights.com/recruit.html

2 http://ameblo.jp/studioaquarius/entry-11473277532.html

3 http://jp.perfectcorp.com/\#ymk
